# Protective Effect of NGR1 against Glutamate-Induced Cytotoxicity in HT22 Hippocampal Neuronal Cells by Upregulating the SIRT1/Wnt/*β*-Catenin Pathway

**DOI:** 10.1155/2021/4358163

**Published:** 2021-12-15

**Authors:** Dong Wang, Bibo Gao, Tao Yang, Huiying Sun, Xiaoping Ran, Wen Lin

**Affiliations:** ^1^Department of Neurological Diseases, Ziyang People's Hospital of Sichuan Province, Ziyang, Sichuan 641300, China; ^2^Department of Neurosurgery, First Affiliated Hospital of Kunming Medical University, Kunming, Yunnan 650030, China; ^3^Department of Neurosurgery, The General Hospital of Western Theater Command, Chengdu, Sichuan 610000, China

## Abstract

Notoginsenoside R1 (NGR1) is an active compound isolated from *Panax notoginseng*. Despite the NGR1 having been used as a traditional medicine, little is known about the neuroprotective effects. In this study, we investigate the protective effects of NGR1 against glutamate-induced cytotoxicity in HT22 cells and its possible molecular mechanism. We assessed the toxicity of NGR1 and the protective activity by MTT assay. The levels of oxidative stress indices superoxide dismutase (SOD), glutathione (GSH), and mitochondrial membrane potential (MMP) were measured by the kits. The levels of reactive oxygen species (ROS) and Ca^2+^ concentration were measured by flow cytometry. Furthermore, we determined the expression of mitochondrial dysfunction related protein PINK1, Parkin, silent mating type information regulation 2 homolog-1 (sirtuin 1; SIRT1), and Wnt/*β*-catenin by Western blotting. Here, we discovered that glutamate treatment led to cell viability loss, apoptosis facilitation, Ca^2+^ upregulation, MMP fluorescence intensity downregulation, and ROS generation of HT22 cells. In parallel, expression of Parkin was declined by glutamate. While, NGR1 treatment alleviated all the above phenomena. We further clarified that NGR1 alleviated glutamate-induced oxidative stress, apoptosis, and mitochondrial dysfunction by upregulating SIRT1 to activate Wnt/*β*-catenin pathways. These findings demonstrate that NGR1 alleviated glutamate-induced cell damage, and NGR1 may play a protective role in neurological complications.

## 1. Introduction

Oxidative stress caused by the accumulation of reactive oxygen species (ROS) is related to the development of many neurological complications [1, 2]. Normally, ROS are generated in the mitochondrial respiratory chain. Excessive ROS induces oxidative stress and apoptosis in neuronal cells [3]. Currently, extracting compounds with antioxidant activity and neuroprotective effects from plants is a potential alternative therapy for neurological complications [4], Therefore, the identification of compounds of plants that inhibit oxidative stress in neuronal cells is crucial to protect the nervous system complications.

Notoginsenoside R1 (NGR1) is an active compound isolated from *Panax notoginseng* [5, 6]. It has antioxidant, anti-inflammatory, antiapoptotic, and antitumor effects [7]. Previous studies have confirmed that NGR1 effectively prevents neurological diseases. In addition, increasing studies have shown that NGR1 can prevent the oxidation, inflammation, and other damages of primary neurons, PC12, and other nerve cells. Like, in PC12 neurotoxicity induced by acrylamide, NGR1 protects PC12 cells by upregulating Trx1 [8]. In diabetic peripheral neuropathy, NGR1 acts as a protective agent by promoting the survival of RSC96 cells [9]. However, there are few studies on the protective effect of NGR1 in HT22 cells.

Glutamate is the main excitatory neurotransmitter in the brain and is considered to be one of the initiating factors of neuronal damage [10–12]. In our study, we exposed HT22 cells to glutamate and investigated the possible mechanism of NGR1's protective effect. According to this study, we found that NGR1 alleviated glutamate-induced oxidative stress and apoptosis by upregulating SIRT1 to activate the Wnt/*β*-catenin pathway.

## 2. Materials and Methods

### 2.1. Reagents and Antibodies

L-glutamic acid, notoginsenoside R1 (NGR1), 98% (HPLC), 2′, 7′dichlorodihydrofluorescein diacetate (H2DCFDA), MTT, and nicotinamide were brought from Sigma-Aldrich (St. Louis, MO, USA). Wnt/*β*-catenin agonist SKL2001 (S8302) was brought from Selleck Chemicals (Houston, TX, USA). Superoxide dismutase (SOD), lactate dehydrogenase (LDH), and glutathione (GSH) assay kits were brought from Nanjing Jiancheng Bioengineering Institute (Nanjing, China). Primary antibodies against Parkin, PINK1, SIRT1, Wnt1, *β*-catenin, cyclin D1, GAPDH, and secondary antibodies were all brought from Cell Signaling Technology (New England Biolabs, Ipswich, MA, USA).

### 2.2. Cell Culture and Treatments

Mouse hippocampal HT22 cells were bought from the Procell Cell Bank (Wuhan, China). HT22 cells were cultured in the DMEM with 10% fetal bovine serum (Gibco, Grand Island, NY), supplemented with penicillin-streptomycin (HyClone, Logan, UT, USA) at 37°C with 5% CO_2_ in a humidified incubator. When cell confluency reached 80%, cells were passaged. All experiments were performed on third passage cells and next, treated with 5 mM glutamate and different concentrations of NGR1.

### 2.3. Cell Viability

Cell viability was detected by the MTT assay. We followed the methods of Wang et al. [13]. Briefly, HT22 cells were seeded into 24-well plates at a density of 1 × 10^4^ cells/cm^2^, according to the manufacturer's instructions. Cells were treated with 5 mM glutamate and different concentrations of NGR1 for 24 h. After treatment, MTT solution (50 *μ*L) was added to the cell culture medium at 37°C for 4 h and added DMSO to plates. Absorbance was measured at 570 nm using a microplate reader. The experiment was repeated three times.

### 2.4. Western Blotting Assay [13]

In the HT22 cells after treatment, add RIPA buffer (Invitrogen; USA) to collect total protein, and the concentration of protein was determined by a bicinchoninic acid protein assay kit (Invitrogen; USA). An equivalent of total protein was resolved on SDS-PAGE and transferred to the polyvinylidene fluoride membrane. Next, the membranes were blocked with 5% nonfat milk and probed with the following primary antibodies overnight at 4°C : SIRT1 (1 : 1000), PINK1(1 : 1000), Parkin (1 : 1000), Wnt1 (1 : 1000), *β*-catenin (1 : 1000), and GAPDH (1 : 2000). Then, they were incubated with diluted secondary antibodies (1 : 3000). followed by immunodetection using an enhanced chemiluminescence kit (Beyotime, China) and analyzed with Image *J* software (NIH, Bethesda, MD, USA).

### 2.5. LDH Release Assay

LDH activity was detected by LDH assay kit. Briefly, cells were collected after glutamate and NGR1 treatment, and separate the supernatant; next, cells' supernatant was incubated with 2,4-dinitrophenylhydrazine; the release of LDH was examined by measurement of the absorbance at 450 nm. The experiment was repeated three times.

### 2.6. ROS Assay

HT22 cells in 6-well plates (1 × 10^4^ cells/well) were treated by NGR1, glutamate, or both. 10 *μ*M H2DCFDA was added into cells and reacted for 20 min at 37°C in the dark. HT22 cells were washed with the serum-free medium for three times. The ROS generation was examined by the flow cytometer (Bender Med Systems, CA, USA).

### 2.7. Flow Cytometry

HT22 cells were treated by NGR1, glutamate, or both. Collect cells and incubate with 5 *μ*L FITC-Annexin V and 1 *μ*L PI (Beyotime Biotechnology, China) working solution for 15 min (in dark). Finally, the fluorescence intensity was measured by flow cytometry (Bender Med Systems, CA, USA).

### 2.8. Determination of Mitochondrial Membrane Potential (MMP)

We followed the methods of Parinee et al. [14]. MMP was evaluated using the commercial kit (Cell Signaling, USA). In brief, HT22 cells were treated by NGR1, glutamate, or both, incubation with TMRE fluorescent dye, and the plate was placed in an incubator for 20 min. Then, after the cells were washed in PBS, the samples were measured with a microplate reader at 488 nm excitation and 574 nm emission.

### 2.9. Statistical Analysis

In our study, all experimental data are presented as mean ± standard error of mean. The difference between two groups was compared by using the *t*-test. Statistical analysis was performed using GraphPad Prism 7.0 software. A value of *P* < 0.05 was considered as statistically significant (^*∗∗*^*P* < 0.01; ^##^*P* < 0.01; ^△△^*P* < 0.01; ^$$^*P* < 0.01).

## 3. Results

### 3.1. The Effects of NGR1 on Glutamate-Induced Cytotoxicity in HT22 Cells

**Figure 1:**
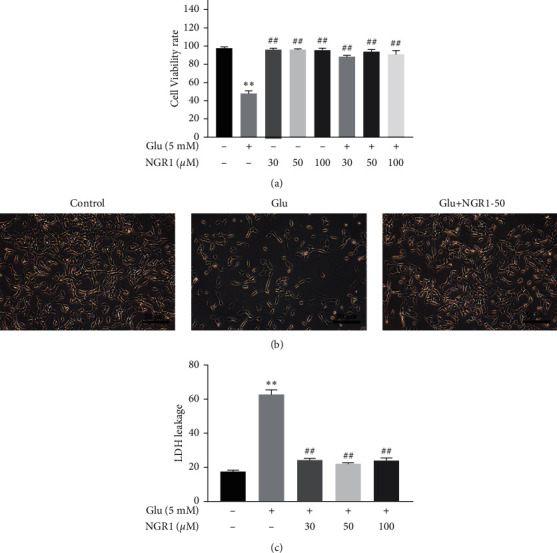
NGR1 alleviated cytotoxicity in the glutamate-induced HT22 cells. HT22 cells viability measured by the MTT assay (48 h) (a). HT22 cells viability observed by microscopic examination (100 ×) (b). The LDH release measured by the LDH assay kit (c). ^*∗∗*^ was considered significant compared to control (^*∗∗*^*P* < 0.01); ## was considered significant compared to glutamate treatment (^##^*P* < 0.01).

The MTT assay was used to evaluate the effect of NGR1 on the viability of HT22 cells. The cell viability assays showed that the viability of HT22 cells was unchanged by NGR1 treatment (Figure 1(a)); after the cells were treated with 5 mM glutamate, cell viability was significantly reduced, and NGR1 treatment alleviated the cell death caused by glutamate (Figure 1(a) and Figure 1(c)). Based on the results of the MTT assay, we choose 50 *μ*M NGR1 as optimum dosage for the following experiments (Glu + NGR1 30 *μ*M: 88.72 ± 3.76%; Glu + NGR1 50 *μ*M: 96.48 ± 2.17%; Glu + NGR1 100 *μ*M: 91.66 ± 2.85%). Simultaneously, treatment with 50 *μ*M NGR1 significantly inhibited Glu-induced LDH leakage (Figure 1(b)). These changes indicated that NGR1 might alleviated the cell death induced by glutamate.

### 3.2. NGR1 Alleviated Glutamate-Induced Apoptosis and Oxidative Stress

**Figure 2:**
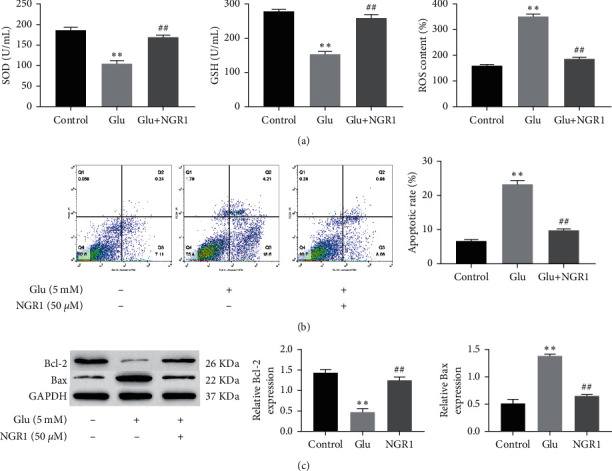
NGR1 alleviated apoptosis and oxidative stress in the glutamate-induced HT22 cells. The levels of SOD and GSH were measured by assay kits. The level of ROS measured by flow cytometry (a). The apoptotic ratio detected by flow cytometry (b). The expressions of Bcl-2 and Bax in cells measured by Western blotting (c). ^*∗∗*^ was considered significant compared to control (^*∗∗*^*P* < 0.01); ## was considered significant compared to glutamate treatment (^##^*P* < 0.01).

We assessed three oxidative stress-associated indicators and levels of Bcl-2, Bax, and apoptosis rate. Our preliminary results showed that treatment with 50 *μ*M NGR1 significantly increased the SOD and GSH content. The levels of ROS were decreased with the treatment of NGR1 (Figure 2(a)). Simultaneously, 50 *μ*M NGR1 significantly reduced glutamate-induced apoptosis (Figure 2(b)). We next evaluated the protein expression of Bax and Bcl-2 and found that treatment with NGR1 significantly upregulated Bcl-2 expression and downregulated Bax expression (Figure 2(c)). Thus, NGR1 alleviated apoptosis and oxidative stress in glutamate-induced HT22 cells.

### 3.3. NGR1 Alleviates the Increase of Ca^2+^ and Mitochondrial Dysfunction in HT22 Cells Induced by Glutamate

**Figure 3:**
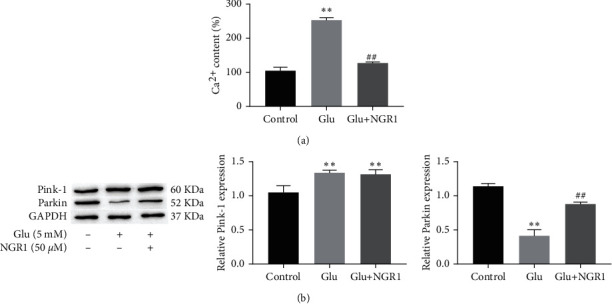
NGR1 alleviates the increase of Ca^2+^ and mitochondrial dysfunction in HT22 cells induced by glutamate. The level of Ca^2+^ measured by flow cytometry (a). The expression of PINK1 and Parkin in HT22 cells measured by Western blotting (b). ^*∗∗*^ was considered significant compared to control (^*∗∗*^*P* < 0.01); ## was considered significant compared to glutamate treatment (^##^*P* < 0.05).

Glutamate induces the further development of oxidative stress in HT22 cells, leads to cell mitochondrial dysfunction, increased Ca^2+^ concentration, and ultimately leads to cellular apoptosis. Next, we wanted to evaluate the effect of NGR1 on mitochondrial dysfunction and Ca^2+^ concentration in glutamate-induced HT22 cells. Our results showed that glutamate significantly decreased the expression levels of Parkin and increased the concentration of Ca^2+^; treatment with 50 *μ*M NGR1 significantly enhanced the expression levels of Parkin and decreased the concentration of Ca^2+^, and PINK1 is upregulated in both glutamate and NGR1 treatments (Figure 3(a) and Figure 3(b)).

### 3.4. NGR1 Alleviates HT22 Cells Apoptosis, Oxidative Stress, Increase of Ca2^+^, and Mitochondrial Dysfunction through Upregulating SIRT1

**Figure 4:**
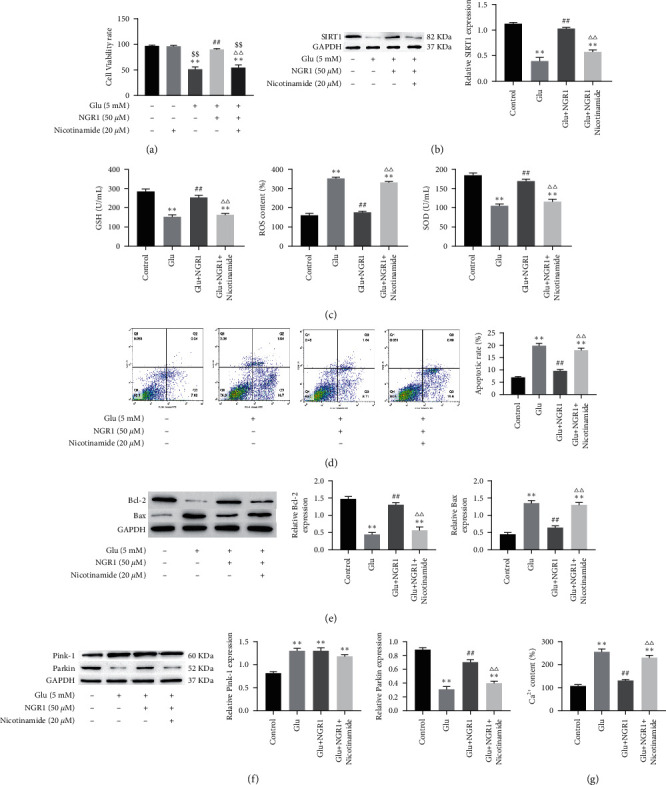
NGR1 alleviates HT22 cells apoptosis, oxidative stress, increase of Ca^2+^, and mitochondrial dysfunction through upregulating SIRT1. HT22 cells viability was measured by the MTT assay (48 h) (a). The expression of SIRT, PINK1, Parkin, Bcl-2, and Bax in cells measured by Western blotting (b), (f), and (e). The levels of SOD and GSH were measured by assay kits. The level of ROS measured by flow cytometry (c). The apoptotic ratio and the level of Ca^2+^ detected by flow cytometry (d) and (g). ^*∗∗*^ was considered significant compared to control (^*∗∗*^*P* < 0.01); ## was considered significant compared to Glu treatment (^##^ < 0.01); △△ was considered significant compared to Glu and NGR1 treatment (^ΔΔ^*P* < 0.01); $$ was considered significant compared to nicotinamide treatment (^$$^*P* < 0.01).

SIRT1 is a protein that is downregulated in neurological complications; to find out whether NGR1 could alleviated cytotoxicity in the glutamate-induced HT22 cells by acting on SIRT1, we use SIRT1 inhibitor nicotinamide (20 *μ*M; for 30 min) to treat cells. The result showed that viability of HT22 cells was unchanged by nicotinamide (20 *μ*M) treatment alone. However, treatment with nicotinamide (20 *μ*M) significantly reduced the cell viability of HT22 cells treated with NGR1 (Figure 4(a)). In addition, Western blotting results showed that SITR1 expression decreased in glutamate-induced HT22 cells, treatment with 50 *μ*M NGR1 significantly enhanced the expression levels of SITR1, and the effect of nicotinamide can be used to reverse this result (Figure 4(b)). Likewise, nicotinamide treatment increased ROS levels and decreased GSH content and SOD activities in cells (Figure 4(c)). Next, we evaluated the apoptosis rate and the protein expression of Bax and Bcl-2; we found that nicotinamide induced cell apoptosis; simultaneously, nicotinamide treatment significantly downregulated Bcl-2 expression and upregulated Bax expression (Figure 4(d) and Figure 4(e)). In addition, nicotinamide treatment decreased the expression levels of Parkin and increased the concentration of Ca^2+^ in HT22 cells, and PINK1 is upregulated in nicotinamide treatment (Figure 4(f) and Figure 4(g)). Thus, NGR1 alleviates HT22 cells apoptosis, oxidative stress, increase of Ca^2+^, and mitochondrial dysfunction through upregulating SIRT1.

### 3.5. Effect of NGR1 on MMP

**Figure 5:**
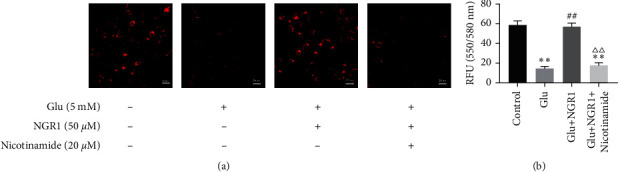
Effect of NGR1 on MMP by upregulating SIRT1. The micrograph of TMRE stained fluorescence was taken with a fluorescence microscope. ^*∗∗*^ was considered significant compared to control (^*∗∗*^*P* < 0.01); ## was considered significant compared to Glu treatment (^##^ < 0.01); △△ was considered significant compared to Glu and NGR1 treatment (^ΔΔ^*P* < 0.01).

We examined the change of MMP in HT22 cells by TMRE staining. The results showed that glutamate significantly reduces the fluorescence intensity of HT22 cells, compared to the control group. Treatment with 50 *μ*M NGR1 significantly enhanced the TMRE fluorescence intensity. In contrast, the effects of NGR1 were reversed by nicotinamide treatment (Figures 5(a) and 5(b)).

### 3.6. NGR1 Activates the Wnt/*β*-Catenin Pathway by Upregulating SIRT1

**Figure 6:**
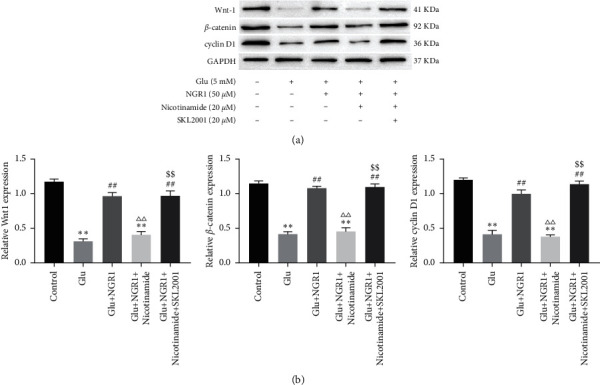
NGR1 activates the Wnt/*β*-catenin pathway by upregulating SIRT1. The expression of Wnt-1, *β*-catenin, and cyclin D1 in HT22 cells measured by Western blotting (a). ^*∗∗*^ was considered significant compared to control (^*∗∗*^*P* < 0.01); ## was considered significant compared to Glu treatment (^##^ < 0.01); △△ was considered significant compared to Glu and NGR1 treatment (^ΔΔ^*P* < 0.01). $$ was considered significant compared to Glu, NGR1, and nicotinamide treatment (^$$^*P* < 0.01).

We measured the expression level of Wnt/*β*-catenin. The results showed that Wnt1, *β*-catenin, and cyclin D1 were significantly downregulated in glutamate-induced cells. After treatment with NGR1, Wnt1, *β*-catenin, and cyclin D1 expressions were significantly increased. However, the expression of Wnt1, c-myc, and cyclin D1 decreased after the nicotinamide treatment. Wnt/*β*-catenin agonist (SKL2001; 10 *μ*M; for 30 min) inhibited the effect of nicotinamide (Figures 6(a) and 6(b)).

## 4. Discussion

Oxidative stress caused by the accumulation of reactive oxygen species (ROS) in nerve cells is associated with neurological complications, including Alzheimer's disease, Parkinson's disease, Huntington's disease, and neuropathic pain [15–17]. The antioxidants are the first defense to detoxify ROS [14]. Identification of effective antioxidant active chemical components from plants or natural products, eliminating active oxygen and maintaining homeostasis, is one of the hotspots of the current research [18, 19]. NGR1 has been reported to treated neurological complications [8, 9]; in addition, NGR1 has anti-inflammatory and antioxidative stress effects [20]. However, the neuroprotective potential of NGR1 against glutamate-induced mediated neurological complications in HT22 cells is unclear. In our present study, we demonstrated the antiapoptosis and antioxidative stress effects of NGR1 on glutamate-induced HT22 cells. We provide evidence that NGR1 alleviates glutamate-induced apoptosis, oxidative stress, and mitochondrial dysfunction, simultaneously restoring Ca^2+^ back to near normal. In addition, SITR1 and Wnt/*β*-catenin is highly expressed under NGR1 treatment. Although previous studies have found the protective effects of SITR1 and Wnt/*β*-catenin in nerves [13], there is no report on the effects of NGR1, SITR1, and Wnt/*β*-catenin. In our study, we demonstrated for the first time that NGR1 prevented glutamate-induced HT22 cells injury by alleviating oxidative stress, the levels of Ca^2+^, and mitochondrial dysfunction through the SIRT1/Wnt/*β*-catenin signaling pathway. Neurons are the functional unit of the nervous system [21–23]. The oxidative stress induced by glutamate will trigger the changes in the structure and function of the neurons and cause the death of neurons [14, 24]. Earlier studies showed that plant extracts and compounds with strong antioxidant activity protected glutamate-induced neuronal death [4, 25]. In the current study, we used mouse-derived hippocampal HT22 cells to study the neuroprotective effect of NGR1 against glutamate-induced cell apoptosis and to explore the neuroprotective mechanism of action. In our study, we found that NGR1 alone is not neurocytotoxic even at high concentrations. However, glutamate alone inhibited HT22 activity, promoted apoptosis, increased levels of LDH, and activated oxidative stress; this is consistent with the results in previous studies [26]. In addition, NGR1 pretreatment significantly protected HT22 cells against glutamate-induced neurocytotoxic by decreasing levels of LDH, inhibiting apoptosis, and oxidative stress. Therefore, these results confirm that NGR1 is an effective antioxidant that exhibits neuroprotective effects by inhibiting the activation of oxidative stress in cells.

Glutamate induces the further development of oxidative stress in HT22 cells, leads to cell mitochondrial dysfunction, increased Ca^2+^ concentration, reduced mitochondrial membrane potential, and ultimately leads to cellular apoptosis [27]. Mitochondrial dysfunction has an integral role in the development of oxidative stress and leads to increased oxidative stress, while the ultrastructure of mitochondria is destroyed, which further exacerbates mitochondrial dysfunction [28]. Plant extracts with antioxidant activity can inhibit glutamate-induced mitochondrial dysfunction and increase in Ca^2+^ concentration in HT22 cells [25]. In this study, we found that NGR1 has an effect on mitochondrial dysfunction and Ca^2+^ concentration. When mitochondria are damaged, activated PINK1 aggregates on the outer mitochondrial membrane and induces mitochondrial autophagy through direct/indirect activated Parkin [29], thereby protecting mitochondrial DNA from reactive oxygen species and stimulating the self-repair process of mitochondria [19]. In this study, we found that NGR1 treatment activates the PINK1/Parkin pathway and upregulates Parkin expression. Simultaneously, NGR1 treatment reduces Ca^2+^ concentration and increases the level of mitochondrial membrane potential in HT22 cells.

As the main regulator of neurological diseases, SIRT1 plays a protective role in neurological diseases by regulating inflammation, oxidative stress, and cell apoptosis [19]. Previous studies demonstrated that activation of SIRT1 alleviates neuronal apoptosis and oxidative injury [30]. In parallel, SIRT1 has been identified as the upstream of the PINK1/Parkin pathway. SIRT1 upregulation mediates mitochondrial autophagy by activating the PINK1-Parkin pathway [31]. In addition, Zhu et al. found that NGR1 can upregulate SIRT1 levels [32]. In the present study, we found that NGR1 significantly increased the expression of SIRT1 in HT22 cells, and the SIRT1 inhibitor nicotinamide weakened the effect of NGR1. Simultaneously, nicotinamide downregulated the expression level of Parkin and weakened the fluorescence intensity of MMP. The Wnt/*β*-catenin signaling system is an important pathway in the development of the CNS [33]. Previous studies have shown that SIRT1, as an activator of the Wnt/*β*-catenin signaling pathway, can increase the transcriptional activity of Wnt1 and *β*-catenin by affecting the stability of c-myc, promote the nuclear transfer of *β*-catenin, and activates the Wnt/*β*-catenin signaling pathway [32, 34]. Moreover, we also observed that the Wnt/*β*-catenin signaling pathway participates in the development process of neurological diseases [35, 36]. In this study, we found that glutamate treatment downregulated the expression of SIRT1, Wnt1, *β*-catenin, and cyclin D1. NGR1 treatment upregulated the expression of SIRT1, Wnt1, *β*-catenin, and cyclin D1. Furthermore, the effects of SIRT1 inhibitor nicotinamide and Wnt/*β*-catenin inhibitor SKL2001 both affect the regulation of SIRT1/Wnt/*β*-catenin by NGR1.

In summary, this study demonstrated that NGR1 plays a neuroprotective role in glutamate-induced cytotoxicity. To the best of our knowledge, this is the first report to show NGR1-protecting HT22 cells against glutamate-induced cytotoxicity by upregulating SIRT1 to activate the Wnt/*β*-catenin pathway. However, one limitation should not be ignored in this study; it is necessary to further verify the mechanism of NGR1 regulation in animal models of neurological diseases.

## Data Availability

The data used to support the findings of this study are available from the corresponding author upon request.
